# HSPB1 facilitates ERK-mediated phosphorylation and degradation of BIM to attenuate endoplasmic reticulum stress-induced apoptosis

**DOI:** 10.1038/cddis.2017.408

**Published:** 2017-08-31

**Authors:** Donna Kennedy, Katarzyna Mnich, Deepu Oommen, Reka Chakravarthy, Leonardo Almeida-Souza, Michiel Krols, Svetlana Saveljeva, Karen Doyle, Sanjeev Gupta, Vincent Timmerman, Sophie Janssens, Adrienne M Gorman, Afshin Samali

**Affiliations:** 1 Apoptosis Research Centre, Biomedical Sciences, NUI Galway, Galway, Ireland; 2 Peripheral Neuropathy Research Group, University of Antwerp, Antwerpen, Belgium; 3 Institute Born Bunge, Antwerpen, Belgium; 4 Discipline of Physiology, NUI Galway, Galway, Ireland; 5 Discipline of Pathology, School of Medicine, NUI Galway, Galway, Ireland; 6 Unit Immunoregulation and Mucosal Immunology, VIB Inflammation Research Centre, Ghent University, Gent, Belgium; 7 Department of Internal Medicine, Ghent University, Gent, Belgium

## Abstract

BIM, a pro-apoptotic BH3-only protein, is a key regulator of the intrinsic (or mitochondrial) apoptosis pathway. Here, we show that BIM induction by endoplasmic reticulum (ER) stress is suppressed in rat PC12 cells overexpressing heat shock protein B1 (HSPB1 or HSP27) and that this is due to enhanced proteasomal degradation of BIM. HSPB1 and BIM form a complex that immunoprecipitates with p-ERK1/2. We found that HSPB1-mediated proteasomal degradation of BIM is dependent on MEK-ERK signaling. Other studies have shown that several missense mutations in HSPB1 cause the peripheral neuropathy, Charcot-Marie-Tooth (CMT) disease, which is associated with nerve degeneration. Here we show that cells overexpressing CMT-related HSPB1 mutants exhibited increased susceptibility to ER stress-induced cell death and high levels of BIM. These findings identify a novel function for HSPB1 as a negative regulator of BIM protein stability leading to protection against ER stress-induced apoptosis, a function that is absent in CMT-associated HSPB1 mutants.

B-cell lymphoma-2 (BCL-2) family proteins are key regulators of the intrinsic apoptosis pathway. Activation of the multi-domain pro-apoptotic members of the family, BCL-2-associated X protein (BAX) and BCL-2-antagonist/killer (BAK), causes mitochondrial outer membrane permeabilization (MOMP) and the release of pro-apoptotic factors such as cytochrome *c* into the cytosol.^1^ The outcome of MOMP is cell death due to caspase activation. BAX/BAK activation occurs either directly or indirectly by BCL-2 homology domain (BH) 3-containing proteins (BH3-only proteins),^2, 3^ whose expression is induced in response to stress stimuli.^4, 5, 6^

BCL-2 interacting mediator of cell death (BIM) is a BH3-only protein that is induced by a range of cellular stresses, ultimately causing cell death.^4, 5, 7^ Thus, BIM levels in cells are under strict regulation to avoid unwanted apoptosis. The regulation of BIM levels is multifaceted. It is transcriptionally induced by transcription factors such as FOXO3 and CHOP.^7, 8^ *Bim* mRNA is also post-transcriptionally regulated by microRNAs such as *miR17-92*, which bind to the 3′UTR causing degradation of *Bim* mRNA and/or preventing its translation.^9, 10^ Conversely, certain stresses such as endoplasmic reticulum (ER) stress can repress microRNAs, indirectly increasing BIM levels.^11^ BIM can be phosphorylated by members of mitogen activated protein kinase family; Extracellular signal-regulated kinase 1/2 (ERK1/2)-mediated phosphorylation stimulates BIM degradation via the proteasome,^12, 13^ whereas phosphorylation mediated by c-Jun N-terminal kinase (JNK) increases BIM pro-apoptotic activity.^14^ Recently, a deubiquitinase was identified that counteracts ERK-dependent BIM ubiquitination, thus stabilizing BIM.^15^

Heat shock preconditioning protects cells from stresses that would ordinarily be toxic.^16, 17^ These effects are mediated by inducible heat shock proteins (HSPs), including HSPB1, which is a potent inhibitor of apoptosis signaling by diverse cellular stressors.^18, 19, 20, 21, 22^ HSPB1 can indirectly inhibit BAX activation/oligomerization and MOMP induction to reduce cytochrome *c* release from the mitochondria.^20, 23^ Mutations in HSPB1 are associated with progressive degeneration of peripheral nerves in the inherited peripheral neuropathies Charcot-Marie-Tooth disease type 2F (CMT2F) and distal hereditary motor neuropathy (dHMN).^24^ There is strong evidence that cellular stress such as ER stress features in certain forms of CMT^25^ highlighting the importance of understanding how HSPB1 regulates this.

Here we show that overexpression of HSPB1 confers protection against apoptosis triggered by ER stress by enhancing the proteasomal degradation of BIM. This effect was dependent on ERK1/2-mediated phosphorylation of BIM. Furthermore, we show that HSPB1 and BIM form a complex with phospho-ERK1/2 that mediates BIM degradation. In contrast to the wild-type HSPB1 protein, HSPB1 variants with CMT-related mutations (S135F, R127W, R136W and T151I) failed to protect against ER stress and were associated with a pronounced increase in BIM levels. Taken together, our data provide another facet to our understanding of how HSPB1 protects upstream of MOMP during apoptosis and show that expression of HSPB1 with CMT-related mutations exacerbates ER stress in cells.

## Results

### HSPB1 overexpression attenuates ER stress-induced intrinsic apoptosis

**Figure 1:**
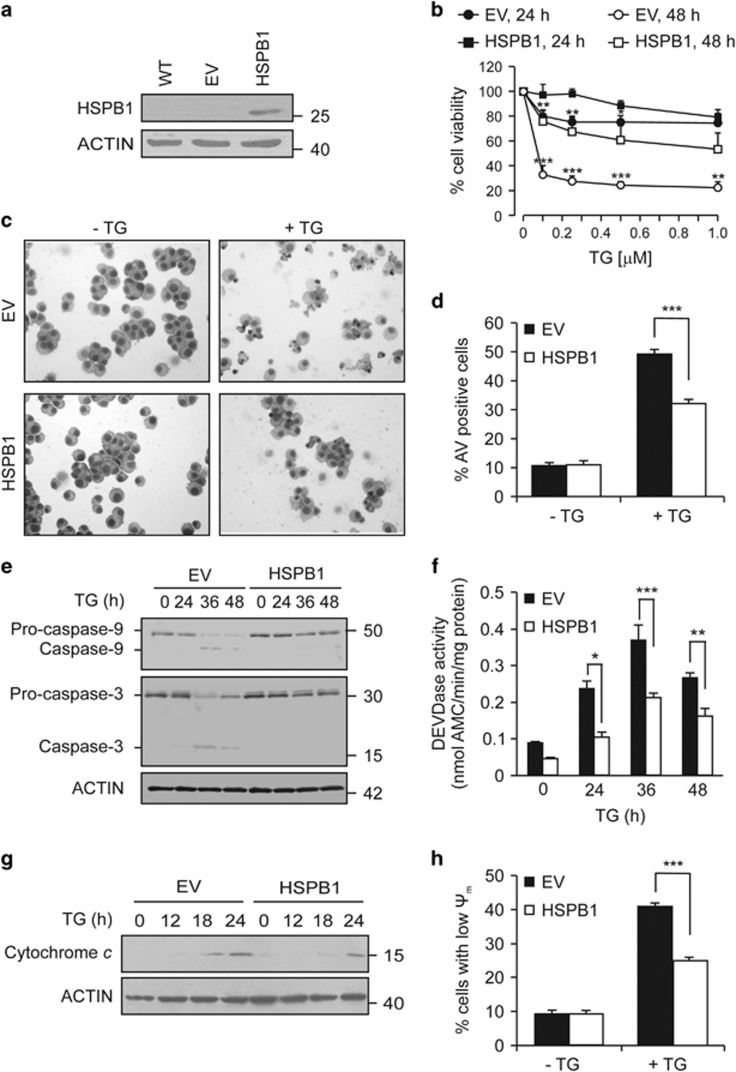
HSPB1 overexpression attenuates ER stress-induced apoptosis. (**a**) Protein extracts of wild-type (WT), empty vector (EV) and HSPB1 PC12 were immunoblotted for HSPB1 and ACTIN (*n*=3). (**b**) EV and HSPB1 PC12 cells were treated with the indicated concentration of TG. Cell viability was assessed by MTT (3-(4,5-dimethylthiazol-2-yl)-2,5-diphenyltetrazolium bromide) assay 24 h and 48 h after treatment (*n*=4). (**c**,**d**) EV and HSPB1 PC12 were treated with vehicle or with 0.25 *μ*M TG for 48 h. Representative bright-field microscopy images of hematoxylin and eosin stained cells are presented (*n*=3) (**c**). The percentage of cell death was determined by flow cytometry-based measurement of Annexin V positivity (*n*=8) (**d**). (**e**–**g**) EV and HSPB1 PC12 cells were treated with a vehicle or 0.25 *μ*M TG for the indicated time. Lysates were immunoblotted for full and cleaved caspase-9 and -3. ACTIN was used as loading control (*n*=3) (**e**). DEVDase activity was measured in whole lysates from cells treated with 0.25 *μ*M TG for the indicated time (*n*=8) (**f**). Release of cytochrome *c* into cytosol was monitored by immunoblotting. ACTIN was used as a loading control (*n*=3) (**g**). (**h**) Loss of mitochondrial membrane potential (ΔΨm) was determined by flow cytometry-based measurement of the percentage of TMRE positive EV and HSPB1 cells after treatment with a vehicle or 0.25 *μ*M TG for 48 h (*n*=3). Data are representative or average±S.E.M. of indicated number of independent biological replicates. Significance was determined using two-way ANOVA followed by Bonferroni’s *post hoc* analysis, with *P*<0.05 being considered significant and annotated by *P<0.05, **P<0.001, ***P<0.0001

We showed previously that heat shock preconditioning protected cells from ER stress-induced apoptosis.^17^ To investigate the role of HSPB1 in that protection, we compared the effect of the two classical ER stress inducers, thapsigargin (TG) and tunicamycin (TM), on PC12 cells stably expressing empty vector (EV) pcDNA3.1 or a vector carrying the full-length human *HSPB1* cDNA sequence (Figure 1a). Treatment of EV cells with increasing concentrations of TG resulted in a significant reduction in cell viability that was ameliorated in cells expressing HSPB1 (Figure 1b). TG-treated EV cells displayed morphological characteristics of apoptosis including cell shrinkage, chromatin condensation and plasma membrane blebbing, features which were attenuated in cells overexpressing HSPB1 (Figure 1c). We confirmed that HSPB1 protects against apoptosis by measuring Annexin V staining (Figure 1d), monitoring pro-caspase-9 and pro-caspase-3 processing (Figure 1e), and caspase-3/-7 activity (DEVDase assay) (Figure 1f). Similarly, caspase processing and activation, induced by TM, were also attenuated in presence of HSPB1 (Supplementary Figure 1a and b). ER stress-induced death is primarily via the intrinsic apoptosis pathway.^26^ We found that HSPB1 reduced the loss of Δψm and cytochrome *c* release into the cytosol of TG-treated cells compared to EV counterparts (Figures 1g and h). Collectively, these results indicate that HSPB1 acts upstream of MOMP to attenuate ER stress-induced apoptosis.

We confirmed UPR activation by TG in the presence and absence of HSPB1 by induction of HSPA5 (also called GRP78), CHOP upregulation, PERK activation (determined by PERK upshift) and eIF2*α* phosphorylation (Supplementary Figure 2a and b).

### HSPB1 downregulates BIM to attenuate ER stress-induced apoptosis

**Figure 2:**
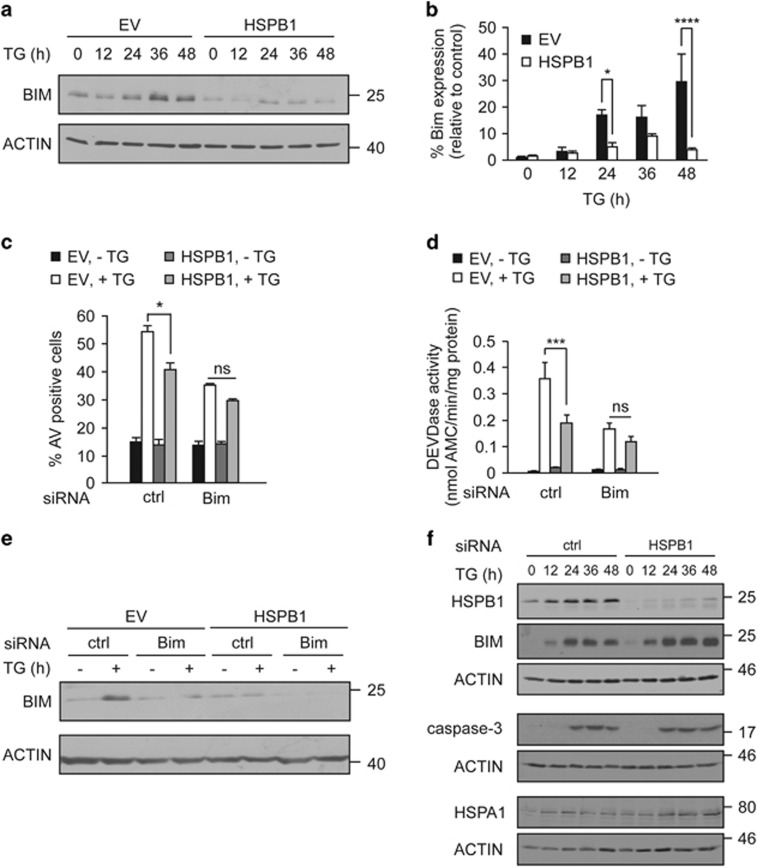
HSPB1 overexpression protects in a BIM-dependent manner. (**a**, **b**) Cells expressing EV or HSPB1, were treated with a vehicle or 0.25 *μ*M TG for up to 48 h. Lysates were immunoblotted for expression of BIM and ACTIN (*n*=3) (**a**). Densitometric and statistical analysis of BIM expression was carried out, and normalized to loading control (**b**). (**c–e**) EV and HSPB1 cells were transfected with *Bim* siRNA or a non-targeting control siRNA (ctrl). The percentage of Annexin V positive cell was analyzed in cells treated with TG for 48 h (*n*=4) (**c**). DEVDase activity was measured in lysates from cells treated with TG for 36 h (*n*=4) (**d**). Protein lysates from cells treated with TG for 48 h were immunoblotted for BIM and ACTIN (*n*=2) (**e**). (**f**) Wild-type PC12 cells were transfected with rat *HspB1* siRNA or a non-targeting control siRNA (ctrl) and treated with a vehicle or 0.25 *μ*M TG 24 h later for up to 48 h. Lysates were immunoblotted for HSPB1, BIM, cleaved caspase-3 and HSPA1. ACTIN was used as a loading control (*n*=2). Data are representative or the average±S.E.M. of indicated number of independent biological replicates. Significance was determined using Two-way ANOVA followed by Bonferroni’s *post hoc* analysis, with *P*<0.05 being considered significant and annotated by *P<0.05, ***P<0.0001, ****P<0.00001, ns (not significant)

Given that heat shock preconditioning protects against ER stress-induced apoptosis in PC12 cells through HSPA1-independent downregulation of BIM,^17^ we examined the role of HSPB1 to regulate ER stress-induced BIM. Following ER stress there was a time-dependent increase in BIM expression in EV cells, which was significantly reduced in HSPB1 cells (Figures 2a and b; Supplementary Figure 1c and d). These findings were also confirmed using cells transiently overexpressing HSPB1, demonstrating that the effect of HSPB1 on BIM is not due to selection of stably transfected cells for deregulated responses to stress (Supplementary Figure 1e). The significance of BIM downregulation by HSPB1 for protection against ER stress was confirmed in EV and HSPB1-expressing cells transfected with *Bim* siRNA and subjected to ER stress (Figures 2c–e).

Next, we knocked down endogenous *Hspb1* in wild-type PC12 cells using siRNA. As expected, *Hspb1* knockdown resulted in BIM accumulation in cells treated with TG compared with control siRNA-transfected counterparts, confirming that HSPB1 is necessary for downregulation of BIM. However, there was no impact on caspase-3 processing (Figure 2f). We previously demonstrated that HSPA1 overexpression inhibits ER stress-induced apoptosis in PC12 cells independent of BIM downregulation.^17, 27^ Given that HSPB1 is induced by ER stress, we checked if HSPA1 is also elevated and found that TG-induced expression of both HSPB1 and HSPA1 (Figure 2f and Supplementary Figure 2c–e). That indicates that induction of HSPs is a general response of PC12 cells to ER stress. We also observed a slight increase in HSPA1 protein levels upon depletion of HSPB1, suggesting a compensatory upregulation of other HSPs in the absence of HSPB1 (Figure 2f), which could further contribute to regulation of caspase-3 processing downstream of BIM.^28, 29^

### Cells expressing HSPB1 with CMT-associated mutations are not protected and express high levels of BIM

**Figure 3:**
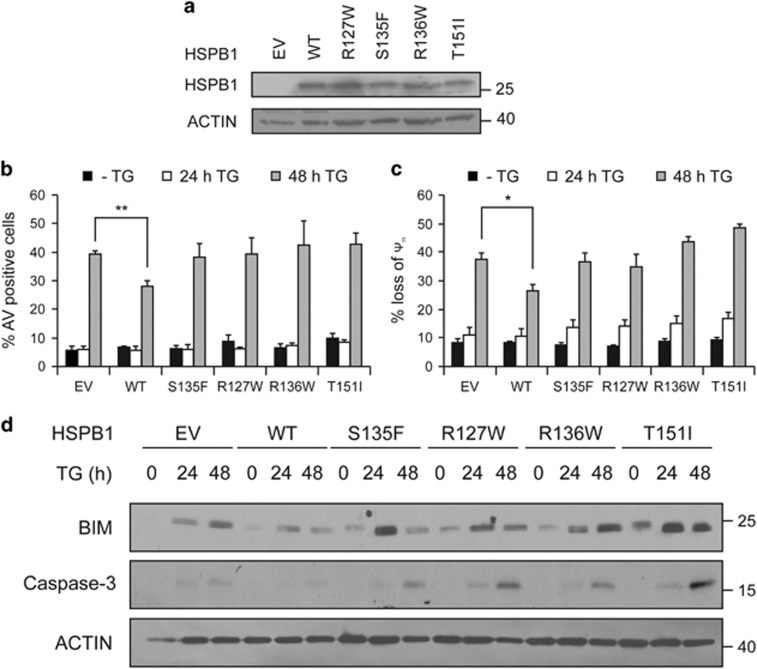
Cells expressing mutants of HSPB1 fail to attenuate ER stress-induced apoptosis and express high levels of BIM. (**a**) Protein lysates from PC12 cells expressing empty vector (EV), wild-type HSPB1, mutant HSPB1 S135F, R127W, R136W and T151I were immunoblotted for HSPB1 and ACTIN (*n*=2). (**b–d**) EV, wild-type HSPB1, S135F, R127W, R136W and T151I PC12 cells were treated with 0.25 *μ*M TG for 24 and 48 h. The percentage of apoptotic cells was determined by flow cytometry-based measurement of Annexin V positivity (*n*=4) (**b**). Loss of mitochondrial membrane potential (ΔΨm) was determined by flow cytometry-based measurement of the percentage of TMRE positive EV and HSPB1 cells after treatment with a vehicle or 0.25 *μ*M TG for 24 and 48 h (*n*=3) (**c**). Lysates were immunoblotted for BIM, cleaved caspase-3 and ACTIN (*n*=3) (**d**). Data are representative or average±S.E.M. of indicated number of independent biological replicates. Significance was determined using Two-way ANOVA followed by Bonferroni’s *post hoc* analysis, with *P*<0.05 being considered significant and annotated by *P<0.05, **P<0.001

Mutations in HSPB1 are associated with disease progression and degeneration of peripheral neurons in CMT neuropathy, but the mechanism remains obscure. We therefore examined the ability of CMT-associated HSPB1 mutants to protect against ER stress-induced apoptosis and to downregulate BIM. We generated PC12 cell lines stably expressing EV plasmid, wild-type HSPB1 (WT) or HSPB1 with CMT-related point mutations (S135F, R127W, R136W or T151I) (Figure 3a). These HSPB1 mutants were chosen because they are located in the *α*-crystallin domain of HSPB1, previously identified as a ‘hot-spot’ in relation to small HSP (sHSP)-associated neuropathies,^30^ and reported to cause CMT.^31^ Although cells overexpressing WT HSPB1 were significantly resistant to ER stress, functional analysis revealed that all mutants failed to protect cells from TG-induced apoptosis (Figures 3b–d), exhibiting no difference in Annexin V staining (Figure 3b), Δψm (Figure 3c) or caspase-3 cleavage (Figure 3d) between TG-treated cells overexpressing HSPB1 mutants and EV controls. Interestingly, whereas WT HSPB1 reduced levels of BIM in stressed cells, the HSPB1 mutants did not prevent BIM accumulation in TG-treated cells (Figure 3d).

### HSPB1 enhances the proteasomal degradation of BIM

**Figure 4:**
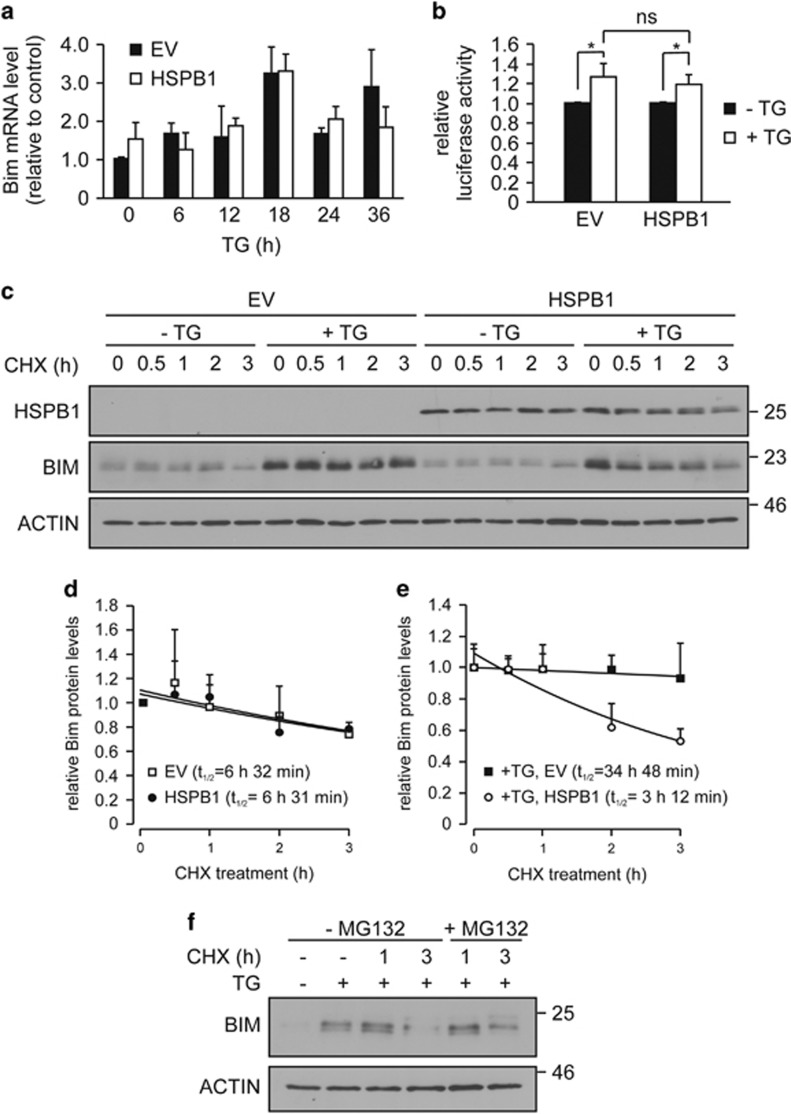
The reduced expression of BIM in HSPB1 cells undergoing ER stress is due to proteasomal degradation. (**a**) RT-qPCR analysis of *Bim* mRNA expression in EV and HSPB1 cell treated with 0.25 *μ*M TG for up to 36 h. Values were normalized to *Gapdh*. Data are average±S.E.M. of three independent biological repeats. (**b**) PC12 cells were transfected with *Bim* 3'UTR reporter or control plasmid and treated with TG for 24 h. Values were normalized to *Firefly* luciferase activity and expression relative to control plasmid is shown. Data are average±S.E.M. of three independent biological repeats. (**c–e**) PC12 cells were treated with a vehicle or with TG for 18 h, followed by 10 *μ*M cycloheximide (CHX) treatment for up to 3 h. Cell lysates were immunoblotted for BIM and ACTIN (*n*=3) (**c**). Densitometric analysis of BIM expression in the absence (**d**) and presence of TG (**e**) was normalized to ACTIN and expressed relative to proper control. Exponential trend lines were fitted through the data points in Word Excel, and the equations were used to calculate half-lives. Values are the average±S.D. of three independent biological replicates. (**f**) HSPB1 PC12 cells were pre-treated with TG for 18 h to induce BIM, followed by treatment with 10 *μ*M CHX and 20 *μ*M MG132 for the indicated time. Lysates were immunoblotted for BIM. ACTIN was used as a loading control (*n*=3). Data are representative of indicated number of independent biological replicates. Significance was determined using two-way ANOVA followed by Bonferroni’s *post hoc* analysis, with *P*<0.05 being considered significant and annotated by *P<0.05. ns (not significant)

To determine the mechanism of BIM downregulation by HSPB1, we first assessed if it occurred at the transcriptional level. *Bim* mRNA induction in response to TG treatment was not affected by HSPB1 overexpression (Figure 4a), indicating a post-transcriptional mechanism for BIM regulation by HSPB1. We utilized a *Bim* 3′UTR reporter plasmid to determine the effect of HSPB1 on regulation of BIM expression at the post-transcriptional level. This luciferase reporter construct spans 1038 nucleotides from bases +2123 to +3160 of the *Bim* 3′UTR that contains binding sites for many microRNAs including members of the miR-106b-25 cluster, key microRNAs involved in the regulation of *Bim* mRNA translation during ER stress.^11^ As expected, TG caused an increase in luciferase activity of the reporter construct (Figure 4b).^11^ Overexpression of HSPB1 did not affect the activity of the *Bim* 3′UTR construct (Figure 4b), demonstrating that HSPB1 neither binds to the *Bim* 3′UTR nor upregulates microRNAs belonging to miR-106b-25 cluster to inhibit *Bim* mRNA translation and/or stability.

Degradation of BIM by the proteasome is one of the well-established mechanisms regulating BIM protein levels.^32^ Given that HSPB1 has been reported to mediate proteasomal degradation of certain client proteins,^33, 34^ we speculated that HSPB1 might increase BIM protein turnover. To test this, we monitored the half-life of BIM in the presence of mRNA translation inhibitor, cycloheximide (CHX) (Figure 4c). The rate of decay of basal levels of BIM was comparable in EV and HSPB1 cells (Figures 4c and d). In EV-expressing cells treated with TG, BIM’s half-life was prolonged from 6.5 h to 34.8 h (Figures 4c and e). This supports a previous report showing that BIM is stabilized during ER stress.^7^ Overexpression of HSPB1 markedly accelerated BIM turnover upon ER stress, and reduced BIM protein half-life to 3.2 h (Figures 4c and e). Furthermore, HSPB1-mediated BIM decay was blocked by proteasomal inhibition with MG132 (Figure 4f). These results demonstrate that the HSPB1-dependent reduction in BIM levels under conditions of ER stress is due to increased proteasomal degradation.

### HSPB1 enhances ERK1/2 phosphorylation and BIM degradation and forms an HSPB1-ERK1/2- BIM complex

**Figure 5:**
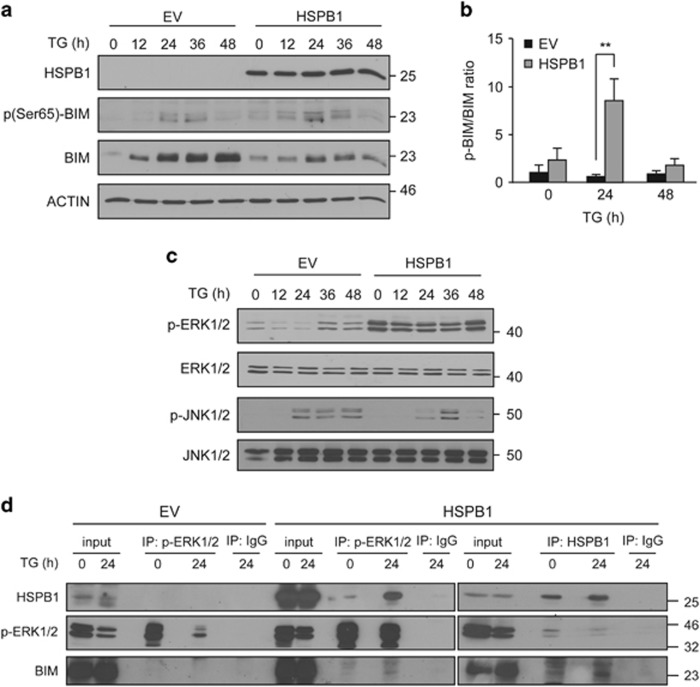
HSPB1 forms complex with p-ERK1/2 and BIM. (**a–c)** EV and HSPB1 PC12 cells were treated with 0.25 *μ*M TG for the indicated time. Lysates were immunoblotted for HSPB1, p-BIM, BIM and ACTIN (*n*=3) (**a**). Densitometric and statistical analysis of relative p-BIM expression in EV and HSPB1 cells was carried out and normalized relative to total BIM (**b**). Cell lysates were immunoblotted for p-ERK1/2 and ERK1/2, p-JNK1/2 and JNK1/2 (*n*=3) (**c**). (**d**) HSPB1 PC12 cells were treated with TG for 24 h followed by immunoprecipitation with control IgG antibody or an antibody specific to HSPB1 or p-ERK1/2. Immune complexes were analyzed by immunoblotting for HSPB1, p-ERK1/2 and BIM (*n*=3). Data are representative or average±S.E.M. of indicated number of independent biological replicates. Significance was determined using two-way ANOVA followed by Bonferroni’s *post hoc* analysis, with *P*<0.05 being considered significant and annotated by **P<0.001

Proteasomal degradation of BIM is mediated by ERK1/2-dependent phosphorylation on Ser69 in human (Ser65 in rodents).^35^ Immunoblotting revealed an increase in BIM phosphorylation at Ser65 in EV and HSPB1 cells treated with TG (Figure 5a). Owing to lower levels of total BIM in HSPB1 cells, there was a significantly higher ratio of phosphorylated BIM to total BIM in HSPB1 cells treated with TG for 24 h compared with EV counterparts (Figure 5b). Furthermore, the basal level of ERK1/2 phosphorylation was significantly elevated upon overexpression of HSPB1 (Figure 5c). HSPB1-overexpressing cells maintained higher levels of p-ERK1/2 compared with EV cells when exposed to ER stress, even though phosphorylation of ERK1/2 transiently declined at 12 and 24 h post-TG treatment and returned to baseline levels after 48 h (Figure 5c). In neuronal cells, BIM can also be phosphorylated by JNK on Ser65.^14, 32^ Here we show that ER stress induced JNK1/2 phosphorylation, which was attenuated at 48 h in HSPB1 cells (Figure 5c).

Our data point to sustained ERK phosphorylation, accompanied by increased BIM degradation in HSPB1 cells. As HSPB1 physically interacts with many of its clients to modulate their activity or half-life,^36, 37, 38^ we hypothesized that HSPB1 facilitates BIM degradation by enhancing ERK1/2 and BIM interaction. Co-immunoprecipitation demonstrated that both p-ERK1/2 and BIM interact with HSPB1 independently of ER stress in HSPB1 cells (Figure 5d) but not in EV cells, thus confirming that HSPB1 is important for complex formation (Figure 5d). A reduction in p-ERK1/2 pull-down was observed in cells treated with TG (Figure 5d), which was probably due to lower levels of p-ERK1/2 (compare input lanes with and without TG treatment, Figure 5d). Reciprocal immunoprecipitation of p-ERK1/2 in HSPB1-overexpressing cells demonstrated co-immunoprecipitation of both BIM and HSPB1 (Figure 5d). The co-immunoprecipitation of HSPB1 with p-ERK1/2 was much higher under ER stress conditions (Figure 5d). This suggests that a proportion of p-ERK1/2 available in cells forms a complex with HSPB1 and BIM, but that in cells treated with TG more of the available p-ERK1/2 interacts with HSPB1. These indicate that in HSPB1 cells HSPB1 interacts with p-ERK1/2 and BIM.

### HSPB1-mediated degradation of BIM is dependent on MEK1/2-ERK1/2 signaling

**Figure 6:**
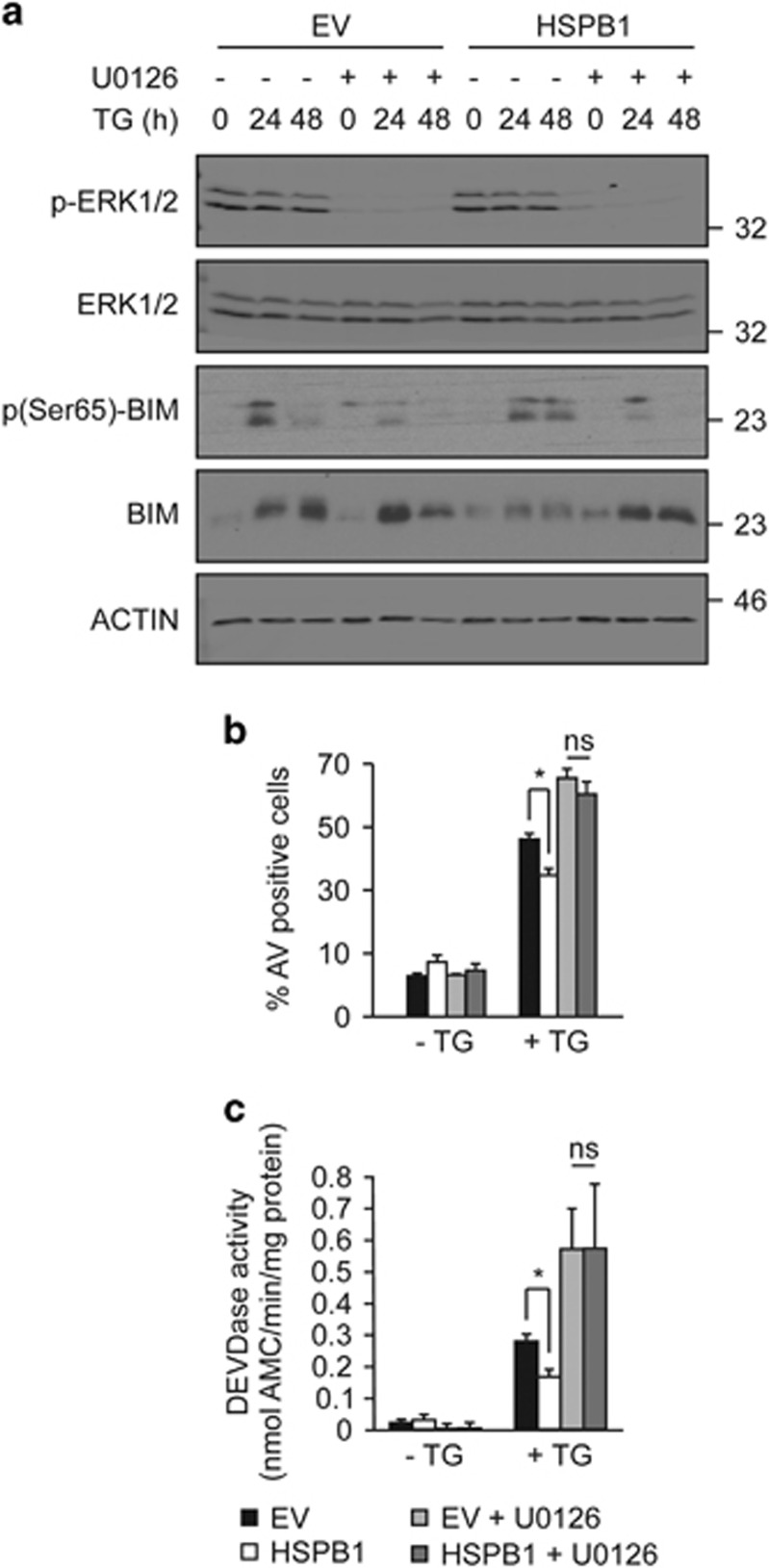
HSPB1-mediated ER stress-induced apoptosis is dependent on the MEK1/2-ERK1/2 pathway. (**a**) EV and HSPB1 cells were treated with 10 *μ*M U0126 for 1 h following TG treatment for 24 and 48 h. Lysates were immunoblotted for BIM, p-BIM, p-ERK1/2 and ERK1/2. ACTIN was used as a loading control. Data shown are representative of three independent biological repeats. (**b**,**c**) EV and HSPB1 PC12 cells were pre-treated with 10 *μ*M U0126 for 1 h before TG treatment for 48 h. The percentage of apoptotic cells were determined by flow cytometry-based measurement of Annexin V positivity (**b**), and DEVDase activity (**c**). Values are average±S.E.M. of three independent biological repeats. Significance was determined using Two-way ANOVA followed by Bonferroni’s *post hoc* analysis, with *P*<0.05 being considered significant and annotated by *P<0.05

To further investigate a role for ERK1/2 in HSPB1-dependent BIM degradation we used a pharmacological approach to inhibit MEK1/2, the upstream kinase that activates ERK1/2.^39^ Treatment of cells with U0126 efficiently blocked ERK1/2 phosphorylation (Figure 6a). There was notable accumulation of BIM and reduction in BIM phosphorylation in HSPB1 cells undergoing ER stress in which MEK1/2 was blocked (Figure 6a). These data indicate that HSPB1-mediated BIM degradation is dependent on MEK/ERK signaling.

We next investigated the role of ERK signaling in the protection afforded by HSPB1 against ER stress-induced apoptosis. Although HSPB1 cells were more resistant to ER stress (Figures 6b and c), the presence of U0126 abrogated HSPB1-mediated resistance to apoptosis, as determined by Annexin V positivity and DEVDase activity (Figures 6b and c). Collectively, these data demonstrate that HSPB1 sustains phosphorylation of ERK1/2 that in turn phosphorylates BIM on Ser65. Further, this signaling event is critical for proteasomal degradation of BIM and for cell survival under conditions of ER stress.

## Discussion

**Figure 7:**
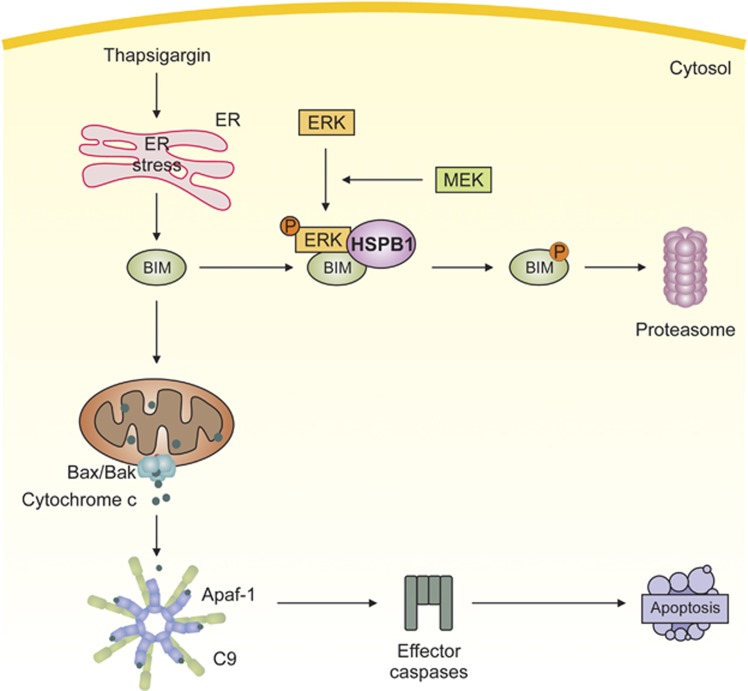
Schematic representation of HSPB1-mediated protection against ER stress-induced apoptosis. Induction of ER stress causes BIM-dependent activation of the mitochondrial or intrinsic apoptosis pathway, which stimulates cytochrome *c* release from mitochondria that leads to apoptosome formation and consequently caspase activation and apoptosis. Expression of HSPB1 causes formation of a protein complex comprising HSPB1, phospho-ERK1/2 and BIM. Phosphorylation of BIM by ERK1/2 targets it for proteasomal degradation

Here, we report for the first time that HSPB1 can regulate the stability of BIM protein by targeting it for proteasomal degradation. This phosphorylation-dependent degradation of BIM, is mediated by ERK1/2, which is supported by the observation that pharmacological inhibition of ERK1/2 activation abrogated HSPB1-mediated BIM protein turnover and sensitized cells to ER stress. Collectively, these data suggest a novel mechanism by which HSPB1 can inhibit the intrinsic apoptosis pathway, and demonstrates a novel way by which BIM is regulated by HSPB1 during cell stress (Figure 7). This work highlights the importance of HSPB1 in orchestrating protection against stress-induced apoptosis, in particular ER stress.

ER stress is known to engage the intrinsic apoptosis pathway through MOMP.^26^ The importance of BIM in activating MOMP during ER stress has been reported by several groups, including our own.^4, 7, 40^ However, the regulation of ER stress-induced apoptosis by HSPs is not well understood. We have previously shown that heat shock preconditioning, which leads to induction of HSPA1 and HSPB1, can protect against ER stress-induced apoptosis through regulation of BIM.^17^ The regulation of BIM did not involve HSPA1. Previous studies have highlighted protective effects of HSPB1 downstream of the mitochondria through direct binding to cytochrome *c* and caspase-3.^41, 42^ Here we demonstrate that HSPB1 can also act upstream of MOMP to avert caspase activation and apoptosis induction in response to ER stress (Figure 1 and Supplementary Figure 1). Other studies have also reported the ability of HSPB1 to modulate apoptotic events upstream of the mitochondria. HSPB1 can stabilize F-ACTIN, which inhibits BID redistribution to the mitochondria and thus cytochrome *c* release.^23^ We show here that targeting BIM for proteasomal degradation represents an additional, novel regulatory mechanism that contributes to HSPB1 inhibition of the intrinsic apoptosis pathway upstream of MOMP.

Our findings underscore the complexity of apoptosis regulation in response to stress. As we do not observe a difference in caspase-3 cleavage upon HSPB1 depletion despite higher levels of BIM (Figure 2f), we speculate that HSPB1 is likely to act in concert with other cellular stress response proteins to regulate apoptosis downstream of BIM. For example, HSPA1, which is induced by ER stress (Supplementary Figure 2c and e), can interfere with apoptosis downstream of MOMP through inhibition of apoptosome formation and caspase-9 activation.^28, 29^ We have shown previously that HSPA1 protects PC12 cells against ER stress-induced apoptosis but does not downregulate BIM.^17, 27^

The regulation of BIM expression is highly complex, incorporating transcriptional induction, post-transcriptional regulation by microRNAs, post-translational modifications such as phosphorylation by ERK leading to BIM ubiquitination and proteasomal degradation or phosphorylation by JNK increasing BIM activity.^43, 44^ HSPB1 has been shown to regulate BIM through translation repression by binding to the *Bim* 3′UTR during oxidative and excitotoxic stress in primary neuronal cultures.^6^ In contrast to this, we did not observe an effect of HSPB1 on *Bim* mRNA levels or on activity at the *Bim* 3′UTR. The reason for the different findings may be due to the use of *Bim* 3′UTR construct that does not overlap with the HSPB1-binding region identified in aforementioned study. We cannot definitively exclude the possibility that HSPB1 acts through an unidentified ER stress-regulated microRNA on a region of the *Bim* 3′UTR that our reporter construct did not include. Nonetheless, the data indicate multiple mechanisms by which HSPB1 can tightly control BIM levels during cellular stress.

To date, only a few client proteins that are targeted to the proteasome in a HSPB1-dependent manner have been identified, including IκBα,^34^ p27^KIP1^,^45^ GATA,^46^ CFTR^47^ and AUF1.^33, 48^ We now expand this list to include a protein that is critically involved in stress-induced apoptosis. It remains unanswered whether HSPB1-mediated targeting of BIM for proteasomal degradation involves BIM ubiquitination. Activation of ERK1/2 signaling has been shown to prime BIM for ubiquitination and subsequent proteasomal degradation.^12^ However, in our studies we did not detect any evidence of BIM ubiquitination. It is interesting to note that ubiquitin-independent proteasomal degradation of BIM driven by ERK1/2 has recently been observed.^49^ Furthermore, HSPB1 can target other client proteins such as CFTR for proteasomal degradation in a ubiquitin-independent manner.^47^ The proteasomal degradation of BIM is dependent on its phosphorylation by ERK1/2.^32^ We observed higher levels of BIM phosphorylation and of active ERK1/2 in HSPB1-overexpressing cells, and furthermore, that HSPB1-mediated degradation of BIM is dependent on active ERK1/2.

HSBP1, BIM and p-ERK1/2 interact to form a complex that was detected in cells. It is possible that HSPB1 acts as a scaffold protein bringing p-ERK1/2 and BIM together to facilitate BIM phosphorylation and proteasomal degradation. HSBP1, like other members of the sHSP family, are ATP-independent, and function by holding misfolded proteins until their proper folding can be catalyzed by ATP-dependent chaperones such as HSPA1.^50^ As BIM is an intrinsically disordered protein,^49^ it is possible that under conditions of stress, HSPB1’s interaction with BIM is prolonged to allow ERK-dependent phosphorylation, or that it holds BIM in a conformation that favors its phosphorylation by ERK1/2.

Although our study focused on the role of HSPB1 in promoting ERK-mediated BIM degradation, other degradation pathways could also play a role. Emerging data suggest that autophagy is part of the global ER stress response.^51^ In human hepatocellular carcinoma cells *hspb1* knockdown can inhibit ER stress-induced autophagy.^52^ Thus, regulation of autophagy might also contribute to the increased survival of HSPB1 cells following ER stress. Whether such a mechanism would also require downregulation of BIM would need further investigation.

Mutations in the *α*-crystallin domain of HSPB1, including R127W, S135F, R136W and T151I, have been linked to CMT type 2 and dHMN.^53^ To date there have been no studies on the response of cells expressing mutant HSPB1 to cellular stress. Given the observed effect of HSPB1 on ERK1/2-mediated degradation of BIM, we explored the response of cells overexpressing mutant HSPB1 to ER stress. Here we show that in contrast to WT HSPB1, the HSPB1 mutants failed to protect against TG-induced apoptosis or to downregulate BIM. In fact, all mutants tested increased BIM levels and sensitized cells to ER stress compared with EV counterparts. HSPB1 R127W, S135F and R136W mutants exhibit increased monomerization and increased chaperone activity compared with the wild-type protein.^30^ In contrast, T151I mutant HSPB1 does not exhibit altered chaperone activity or increased interaction with client proteins.^30^ This suggests that the regulation of BIM by HSPB1 is independent of its chaperone activity. An alternative explanation is that the interaction between BIM and mutant HSPB1 is prolonged such that BIM degradation is inhibited. However, this does not account for the observation that the highest levels of BIM and of ER stress-induced apoptosis are observed with the T151I mutant. There is still a clear need to fully elucidate the physico-chemical and functional properties of HSPB1 mutant proteins.

HSPB1 undergoes dynamic organization between small and large oligomers ranging up to 1000 kDa.^54^ These dissociate into dimers and tetramers under stress conditions.^55, 56^ This affects HSPB1’s chaperone function and ability to interact with client proteins. Previous studies have shown that, at least in the case of p27^KIP1^, small rather than large oligomers of HSPB1 are required to increase p27^KIP1^ degradation.^45^ As small HSPB1 oligomers are associated with cellular stress this suggests that cellular stress promotes HSPB1 activity regarding proteasomal degradation. We observed that HSPB1 overexpression significantly reduced the half-life of BIM under ER stress but not under normal conditions, suggesting that cellular stress might be required for the effect of HSPB1 on BIM regulation. Indeed, TG caused an increase in abundance of HSPB1 dimers and trimers (Supplementary Figure 3). Our data suggest that under conditions of ER stress HSPB1 interacted more strongly with p-ERK1/2 than under basal conditions. This could indicate reorganization of HSPB1 quaternary structure during ER stress. Understanding how p-ERK1/2 and BIM interact with HSPB1 might provide insights to guide structure-based therapeutic strategies to disrupt or enhance this complex. In recent years, peptide aptamers (PAs) have been developed that positively or negatively modulate the function and oligomeric status of HSPB1.^36^ Such PAs will likely prove to be valuable tools in the generation of chemical modulators of HSPB1, which would have implications for the treatment of several diseases including CMT and other motor neuropathies where HSPB1 mutations are involved in the disease. Activation of ER stress is a salient feature of several neurodegenerative diseases.^57^ In light of our finding that HSPB1 can inhibit ER stress-induced apoptosis through increasing proteasomal degradation of BIM, therapies aimed at increasing this activity may be of value in neurodegenerative diseases in which ER stress has a pathophysiological role.

## Materials and Methods

### Reagents

TG (T9033), TM (T7765), U0126 (U120), CHX (C7698) and MG132 (C2211) were purchased from Sigma-Aldrich (Saint Louis, Missouri, USA). Ac-Asp-Glu-Val-Asp-a-(4-methyl-coumaryl-7-amide) (DEVD-MCA) was from the Peptide Institute (Osaka, Japan, 3171-v).

### Cell culture

Rat pheochromocytoma PC12 cells were obtained from the European Collection of Authenticated Cell Cultures (Salisbury, UK). Cells were routinely tested for mycoplasma contamination and only mycoplasma-free cells were used. Cells were maintained in high-glucose Dulbecco’s Modified Eagle’s medium from Sigma (D6429) supplemented with 10% heat inactivated horse serum, 5% fetal bovine serum, and 1% penicillin/streptomycin (Sigma) at 37 °C, 5% CO_2_ in humidified incubator. Cells were seeded in poly-l-lysine-coated dishes at 4 × 10^4^ cells/cm^2^ density 24 h prior to treatments. TG was used at a concentration of 0.25 *μ*M and TM was used at 2 *μ*g/ml unless otherwise stated.

### Immunoblotting

Cells were lysed in RIPA buffer (50 mM Tris-HCl, pH 8.8, 150 mM NaCl, 0.5% (w/v) sodium deoxycholate, 0.1% (w/v) SDS, 1% (v/v) NP-40) containing protease inhibitors (1 *μ*M phenylmethylsulphonyl fluoride (PMSF), 1 *μ*g/ml Pepstatin, 10 *μ*M Leupeptin, 2.5 *μ*g/ml Aprotinin and 250 *μ*M ALLN) and phosphatase inhibitors (10 mM NaF, 1 mM Na_3_VO_4_). Proteins were separated by SDS-PAGE, transferred to nitrocellulose membranes (GE Healthcare, Germany), which were then incubated in 5% (w/v) non-fat milk dissolved in blocking solution (phosphate-buffered saline containing 0.1% (v/v) Tween 20). Membranes were probed with specific antibodies for: ACTIN (Sigma, A2066, 1 : 5000), p- BIM (CST, Danvers, MA, USA, #4585, 1 : 1000), BIM (Stressgen, Farmingdale, NY, USA, ADI-AAP-330, 1 : 2000), Caspase-3 (CST, #9662, 1 : 1000) Cleaved Caspase-3 (CST, #9664, 1 : 1000), Caspase-9 (CST, #9508, 1 : 1000), p-ERK1/2 (CST, #9101, 1 : 1000), ERK1/2 (CST, #4696, 1:2000), p-JNK (CST, #9255, 1 : 1000), JNK (CST, #9258, 1 : 2000), human HSPB1 (Stressgen, ADI-SPA-803, 1 : 1000), rat HSPB1 (Stressgen, ADI-SPA-801), HSPA1 (Stressgen, ADI-SPA-811), cytochrome *c* (BD Biosciences, San Jose, CA, USA, #556433, 1 : 1000), CHOP (CST, #2895, 1 : 2000), p-eIF2*α* (CST, #3398, 1 : 1000), PERK (CST, #3192, 1 : 1000), HSPA5 (Stressgen, ADI-SPA-826, 1 : 1000). Phospho-specific antibodies were prepared in 5% (w/v) bovine serum albumin in PBS containing 0.1% (v/v) Tween 20. All remaining antibodies were diluted in blocking solution. The horseradish peroxidase-conjugated secondary antibodies were purchased from Jackson Immunoresearch Europe Ltd (UK). The signal was visualized using enhanced chemiluminescence reagent (Perkin Elmer, Waltham, MA, USA, NEL102001EA).

### MTT assay

The mitochondrial metabolic function of cells was assayed by monitoring the conversion of MTT (3-(4, 5-dimethylthiazol-2-yl)-2, 5-diphenyl tetrazonium bromide) (Sigma, M5655) to purple formazan crystals in viable cells. PC12 cells were plated into 96-well plates at 4 × 10^4^ cells/cm^2^ in triplicate. After carrying out an experiment, cells were incubated with 0.5 mg/ml of MTT for 3 h at 37 °C. To stop the reaction and solubilize the formazan crystals 1 volume of 20% (w/v) SDS in 50% (v/v) dimethyl formamide was added and the absorbance was measured at 550 nm by a Wallac 1420 plate-reader with a reference wavelength of 650 nm. Cell viability was expressed as percent of viable cells relative to the control.

### Hematoxylin and eosin staining

Cells were scraped from culture flask and 5 × 10^4^ cells were spun onto glass slides (Shandon Cytospin 3), fixed in methanol for 5 min at room temperature and stained in Harris hematoxylin solution for 5 min. The staining was differentiated by a quick acid wash (25 mM HCl in 70% (v/v) ethanol) and blued in Scott’s water (1% (w/v) MgSO_4_, 0.2% (w/v) NaHCO_3_ in water). The cytosol was stained with Eosin Y for 10 s. Coverslips were placed on slides using DPX mounting medium (Sigma). Cells were observed by light microscopy (Olympus BX51 microscope).

### Annexin V staining

Binding of Annexin V-FITC to externalized phosphatidylserine was used to determine extent of apoptosis in cultures. In brief, cells were trypsinized, suspended in culture medium cells and left to recover for 15 min at 37 °C. Cells were collected by centrifugation at 300 *g* for 5 min. The supernatant was removed and cells were re-suspended in 50 *μ*l binding buffer (10 mM HEPES, pH 7.5, 140 mM NaCl, 2.5 mM CaCl_2_) containing 1 *μ*l of homemade Annexin V-FITC and incubated in the dark on ice for 15 min. Prior to analysis 300 *μ*l of binding buffer was added. A total of 10000 cells per sample were collected using a FACSCalibur flow cytometer (Becton Dickinson, Franklin Lakes, NJ, USA) and analyzed by Cyflogic software.

### Detection of DEVDase activity

Cells were harvested, pelleted by centrifugation at 350 *g*, washed and re-suspended in 50 *μ*l of ice-cold PBS. Each lysate was divided equally into two wells of a microtiter plate and snap-frozen in dry ice. Cell lysates and caspase substrate, Ac-DEVD-MCA, were combined in reaction buffer (100 mM HEPES, pH 7.5, 10% (w/v) sucrose, 0.1% (w/v) CHAPS, 5 mM DTT, and 0.0001% (v/v) NP-40, and 50 *μ*M Ac-DEVD-MCA). The release of fluorescent AMC was monitored at 1 min intervals over 1 h by Wallac Victor 1420 multilabel counter (Perkin Elmer Life Sciences, Waltham, MA, USA) using excitation 355 nm, and emission 460 nm wavelengths at 37 °C. Fluorescent units were converted to nmol of AMC released using a standard curve generated with free AMC and subsequently related to protein concentration.

### Determination of cytochrome c release

Cells were trypsinized and centrifuged at 150 *g* for 5 min at 4 °C. The cell pellet was washed once with PBS. The cells were then lysed using 100 *μ*l cell lysis and mitochondria intact buffer containing (250 mM sucrose and 70 mM KCl in PBS, 0.1 mM PMSF, 1 mM Dithiothreitol, 5 *μ*g/ml Pepstatin, 10 *μ*g/ml Leupeptin, 2 *μ*g/ml Aprotinin and 25 *μ*g/ml Calpain inhibitor 1). Digitonin (10 *μ*l of 20 mg/ml solution) was added to the samples on ice for 5 min and then the cell suspension was centrifuged at 3000 *g* for 10 min at 4 °C. The supernatant was removed and stored as the cytosolic fraction, at −20 °C.

### Changes in mitochondrial transmembrane potential

To measure mitochondrial transmembrane potential (ΔΨm) cells were incubated with the fluorescent probe 10 ng/ml tetramethylrhodamine ethyl ester (Molecular Probes, Eugene, OR, USA, T669) added to the culture media for 30 min at room temperature. A total of 10000 cells per sample were then analyzed on a FACSCalibur flow cytometer (Becton Dickinson).

### Densitometric analysis

Quantitative analysis of immunoblotting results was performed using densitometric analysis with ImageJ software. The protein expression was normalized to the loading control (ACTIN) and expressed relative to untreated sample (otherwise indicated).

### siRNA transfection

The sequences of siRNA (Dharmacon, Lafayette, CO, USA) used in this study included following:

siGENOME SMART pool siRNA against Rat *Bcl2l11* (*Bim*) (M-093533-01-0005):

D-093533-04: 5′-GUAAAUUCUGAGUGUGACA-3′

D-093533-03: 5′-ACGAGUUCAAUGAGACUUA-3′

D-093533-02: 5′-CGAGGAGGGCGUUUGCAAA-3′

D-093533-01: 5′-GGUUAUCUUACAACUGUUA-3′

and a non-targeting control (D-001206-13-00):

D-001206-13-01: 5′- AUGAACGUGAAUUGCUCAA-3′

D-001206-13-02: 5′- UAAGGCUAUGAAGAGAUAC-3′

D-001206-13-03:5′-AUGUAUUGGCCUGUAUUAG-3′

D-001206-13-04: 5′- UAGCGACUAAACACAUCAA-3′

ON-TARGET plus SMART pool siRNA against Rat *HspB1* (L-080155-02-0005):

J-080155-05: 5′-CGAGGCCCGUGCCCAAAUU-3′

J-080155-06: 5′-GGAACAGUCUGGAGCCAAG-3′

J-080155-07: 5′-CUACAUCUCUCGGUGCUUC-3′

J-080155-08: 5′-CCAAAGCAGUCACACAAUC-3′

and a non-targeting control (D-001810-10-20):

5′-UGGUUUACAUGUCGACUAA-3′

5′-UGGUUUACAUGUUGUGUGA-3′

5′-UGGUUUACAUGUUUUCUGA-3′

5′-UGGUUUACAUGUUUUCCUA-3′

siRNAs were re-suspended in RNase and DNase free Sigma water and stored at −80 °C. PC12 cells were transfected with 20 nM of *Bim* siRNA or 40 nM *Hspb1* siRNA using Lipofectamine 2000 (Fermentas, Waltham, MA, USA). The RNA-Lipofectamine complexes were incubated at room temperature for 20 min. The complexes were added drop wise to the cells and incubated for 5 h at 37 °C. Media was changed and 24 h later cells were used for experiments.

### Plasmids

PC12 cells stably expressing wild-type HSPB1, R127W HSPB1, S135F HSPB1, R136W HSPB1 and T151I HSPB1-overexpressing PC12 cells and control EV cells were generated by transfection with empty pcDNA3.1(+) (Invitrogen, Carlsbad, CA, USA) or pcDNA3.1 containing human HSPB1 cDNA sequence. The following primers were used to generate the coding sequence for:

R127W HSPB1: forward 5′-GGCAAGCACGAGGAGTGGCAGGACGAGCAT-3′, reverse 5′-ATGCTCGTCCTGCCACTCCTCGTGCTTGCC-3′

S135F HSPB1: forward 5′-GAGCATGGCTACATCTTCCGGTGCTTCACGCGG-3′, reverse 5′-CCGCGTGAAGCACCGGAAGATGTAGCCATGCTC-3′

R136W HSPB1: forward 5′-CATGGCTACATCTCCTGGTGCTTCACGCGG-3′, reverse 5′-CCGCGTGAAGCACCAGGAGATGTAGCCATG-3′

T151I HSPB1: forward 5′-GGTGTGGACCCCATCCAAGTTTCCTCCTCC-3′, reverse 5′-GGAGGAGGAAACTTGGATGGGGTCCACACC-3′.

The PCR products containing the mutant open reading frames were then recombined into a pcDNA vector, and the sequence was validated by sequencing. Stable cell lines were produced by transient transfection and G418 selection as previously described.^17^ Stable cells lines had been checked for similar expression levels of HSPB1 (Figure 3a).

### RNA extraction and real-time PCR

Total RNA was isolated using Trizol (Invitrogen) according to the manufacturer's instructions. A total of 2 *μ*g of RNA was DNase treated followed by inactivation of DNase by EDTA (Invitrogen). Reverse transcription was carried out with Oligo dT (Invitrogen) using 20 U Superscript II Reverse Transcriptase (Invitrogen). cDNA was diluted 1:6 and mixed with 2 × TaqMan master mix and 20 × TaqMan Gene Expression Assays (Applied Biosystems, Foster City, CA, USA). The cDNA was subjected to 40 cycles of real-time PCR (95 °C for 10 s, 95 °C for 15 s and 60 °C for 60 s) using the forward primer 5′-GTTGTAAGATAACCATTTGCGGG-3′ and reverse primer 5′-GAGGAACCTGAAGATCTGCG-3′ specific for *Bcl2l11* (*Bim*). *Gapdh* (forward 5′-ACCACAGTCCATGCCATC-3′ and reverse 5′-TCCACCACCTGTTGCTG-3′) was used as an internal control.

### Luciferase assay

The *Bim* 3′UTR construct was a gift from Dr. Klaus Rajewsky, Harvard University, USA.^58^ The *Bim* 3′ UTR reporter plasmid containing the putative binding site for members of miR17-92 cluster was cloned into the psiCHECK2 vector (Promega, Madison, WI, USA), downstream the *Renilla* luciferase gene. A psiCHECK2 vector also contains the *Firefly* reporter gene as the intraplasmid transfection normalization reporter. In *Bim* 3′UTR reporter assays, PC12 cells were transfected with 0.75 *μ*g of psiCHECK2 vector or reporter plasmid containing the *Bim* 3′UTR using Lipofectamine 2000 according to the manufacturer’s instructions. Medium was changed 5 h after transfection and treatments were performed 24 h later.

Cells were collected by spinning at 5000 *g* for 3 min using soft acceleration and re-suspended in 50 *μ*l of Dual-Glo Luciferase Reagent (Promega). *Firefly* luciferase and *Renilla* luciferase activities were measured at 560 nm using 10 s luminescence protocol on Wallac plate-reader and then normalized for *Firefly* luciferase activity. Stop and Glo buffer was added between those two measurements.

### Assessment of protein half-life

PC12 cells were treated with TG for 18 h followed by treatment with 10 *μ*M CHX for up to 3 h. Cells were harvested at the indicated time and lysed to obtain protein extracts. Immunoblotting analysis was performed as described previously. Densitometric analysis was carried out and normalized to loading control (ACTIN). Data were fit to a mono-exponential decay curve using Microsoft Excel where untreated or TG-treated samples were set to 1.

### Immunoprecipitation

Immunoprecipitation experiments were performed using the Dynabeads Co-Immunoprecipitation Kit (Life Technologies, Lithuania, 14321D). In brief, on the day of the treatment, the beads were conjugated with HSPB1 or p-ERK1/2 specific antibody or rabbit IgG (Jackson Immunoresearch, 011-000-003) for 18 h at 37 °C. After the indicated time points cells were harvested and lysed in NP-40 lysis buffer (150 mM NaCl, 1% (v/v) NP-40, 10% (v/v) glycerol, 10 mM Tris pH 8) with protease inhibitors (Roche Diagnostics GmbH, Germany, 11873580001) and phosphatase inhibitors (10 mM NaF, 1 mM Na_3_VO_4_) for 1 h at 4 °C. Lysates were centrifuged at 150 *g* for 5 min at 4 °C. Beads were added to the lysate and incubated for 4 h at 4 °C with constant rotation. After the beads were washed, samples were eluted and the 1 × Laemmli buffer was added. Samples were boiled for 5 min at 95 °C, run on the SDS-PAGE gel and analyzed by immunoblotting as described before.

### Assessment of oligomeric status of HSPB1 following ER stress

Cells were treated with 0.25 *μ*M TG, TM for 12 h or heat shocked (as described before)^17^ and left to recover for 2 h. Cells were harvested, pelleted by centrifugation at 350 *g*, and washed in PBS. Cell pellets were lysed in lysis buffer (25 mM HEPES, pH 7.4, 3.3% (v/v) glycerol, 1 mM EDTA, 1 mM DTT, 0.1 mM PMSF) and snap-frozen with liquid nitrogen and thawed at 37 °C. Lysates were chemically cross-linked by incubating with 1 volume of 0.1% (v/v) glutaraldehyde for 40 minutes at 30 ^o^C. The cross-linking reaction was stopped by adding one volume of stopping buffer (1 M Tris-HCl containing 10% (w/v) SDS and 10 mM EDTA). Crossed-linked HSPB1 species were detected by immunoblotting as described before.

### Statistical analysis

Error bars represent means±S.E.M. of independent biological replicates. Significance was determined using two-way ANOVA followed by Bonferroni’s *post hoc* analysis, with *P*<0.05 being considered significant and annotated by *P<0.05, **P<0.001, ***P<0.0001, ****P<0.00001.
