# Supplementary material for: HSPB1 facilitates ERK-mediated phosphorylation and degradation of BIM to attenuate endoplasmic reticulum stress-induced apoptosis
Source: Cell Death Dis. 2017 Aug 31;8(8):e3026–. doi: 10.1038/cddis.2017.408 (PMC5596589; doi:10.1038/cddis.2017.408)
Supplement: Supplementary Information [file cddis2017408x1.pdf]

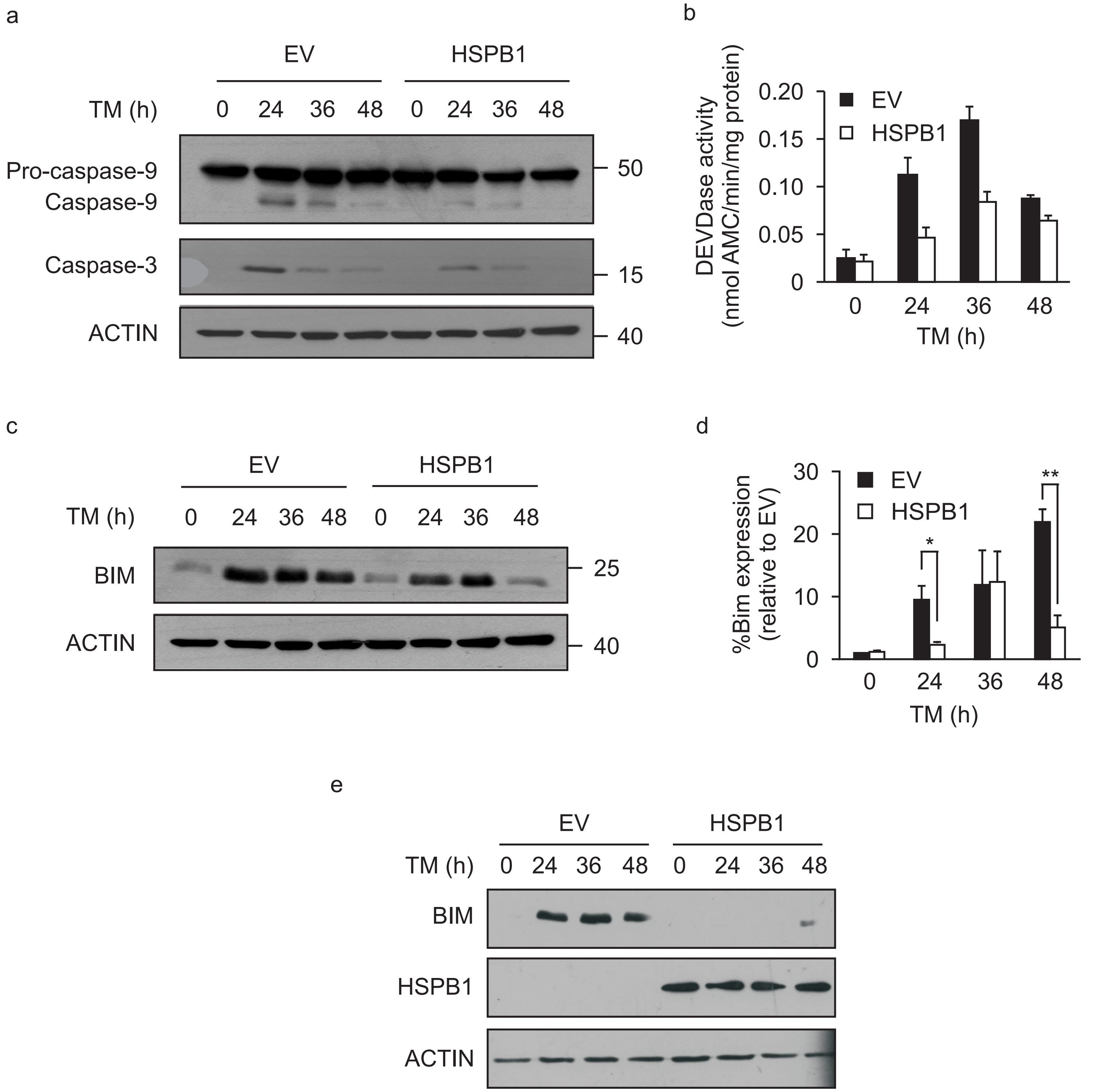

Supplementary Figure 1 HSPB1 attenuates TM-induced apoptosis and downregulates expression of BIM.

(a-d) PC12 cells expressing EV and HSPB1 were treated with vehicle or 2 μg/ml TM for indicated times. Lysates were immunoblotted for full-length and cleaved caspase-9 and cleaved caspase-3. ACTIN was used as a loading control (n=3) **(a)**. DEVDase activity was measured (n=3) **(b)**. Protein lysates were immunoblotted for BIM and ACTIN (n=3) **(c)**. BIM expression normalized to control was determined by densitometric analysis **(d)**. Wild type PC12s were transiently transfected with EV or HSPB1 followed by treatment with TM for the indicated time. Protein lysates were immunoblotted for BIM and ACTIN (n=2) **(e)**. Data is representative or average ± S.E.M. of indicated number of independent biological replicates. Significance was determined using Two-way ANOVA followed by Bonferroni's *post hoc* analysis, with $p<0.05$ being considered significant and annotated by $*P<0.05$, $**P<0.001$.

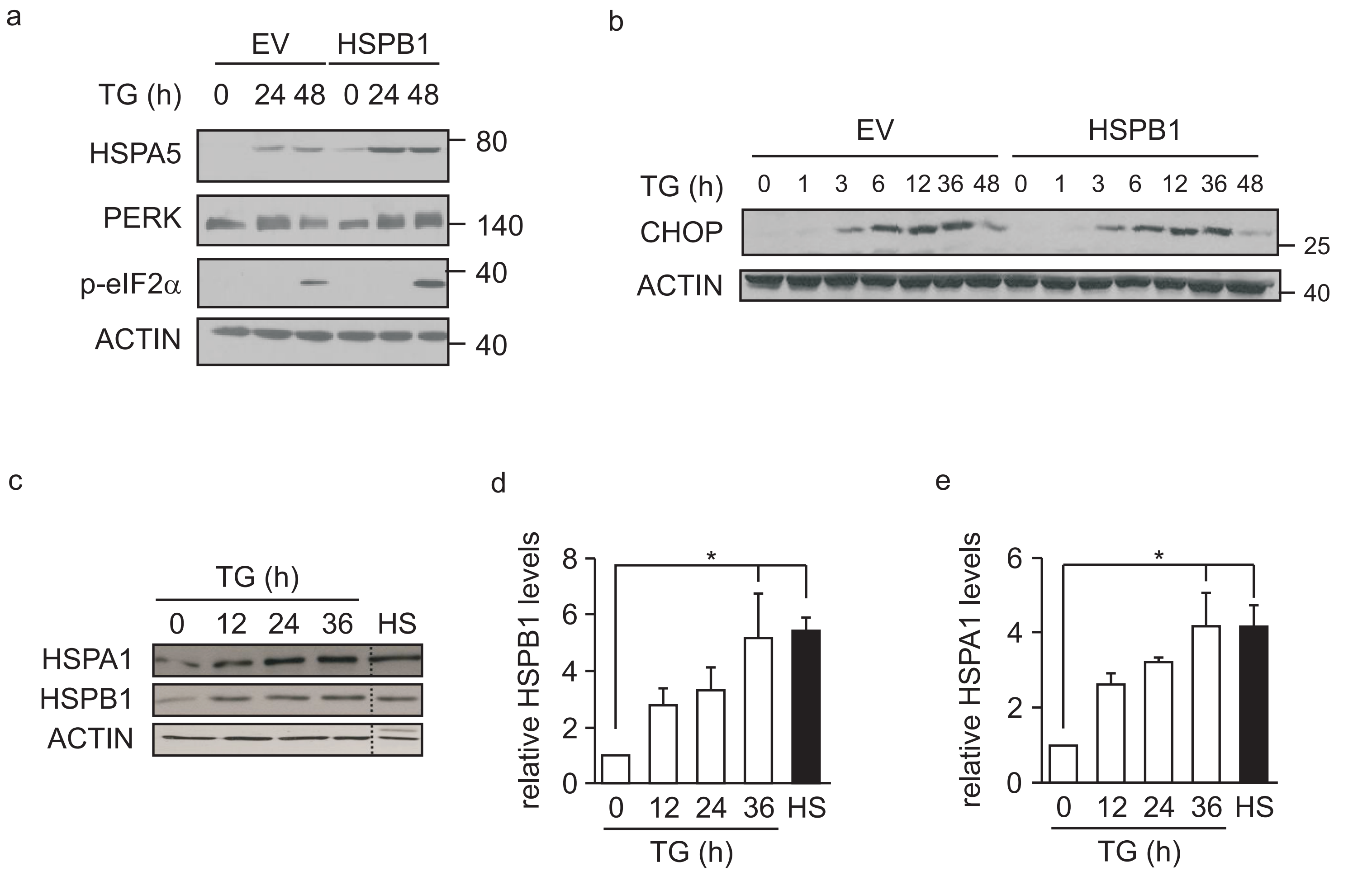

Supplementary Figure 2 Cross-talk between ER stress and heat shock response signaling pathways

(a,b,c) EV and HSPB1 PC12 cells **(a,b)** or wild-type PC12 cells **(c)** were treated with vehicle or 0.25 μM TG for indicated times. Immunoblotting has been performed to determine expression of HSPA5, PERK, p-eIF2α **(a)** CHOP **(b)**, HSPB1 and HSPA1 **(c)**. ACTIN was used as a loading control. Data are representative of three independent biological repeats. **(d,e)** Densitometric and statistical analysis of HSPB1 and HSPA1protein expression following TG treatment of wild-type PC12 cells was carried out and normalized to loading control. Data was normalized to vehicle-treated control.Data is average ± S.E.M. of three independent biological replicates. *$P < 0.05$ One-way ANOVA followed by Tukey's post hoc analysis.

a

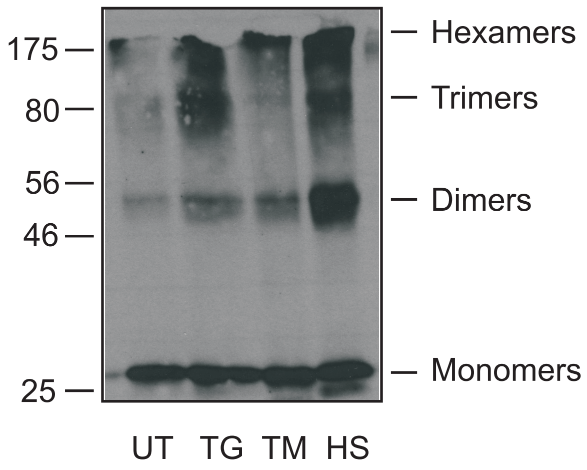

Supplementary Figure 3 ER stress alters an oligomeric state of HSPB1

HSPB1 overexpressing PC12 cells were untreated (UT) or treated with 0.25 μM TG, 0.25 μM TM for 12 h or heat shocked (HS) for 1 h at 42 °C and left to recover for 2 h. Protein lysates cross-linked with glutaraldehyde were immunoblotted for HSPB1. Data is representative of two independent biological repeats.
